# Hypolipidemic mechanism of *Pleurotus eryngii* polysaccharides in high-fat diet-induced obese mice based on metabolomics

**DOI:** 10.3389/fnut.2023.1118923

**Published:** 2023-01-25

**Authors:** Yuanyuan Zhao, Zhen Zhang, Li Wang, Wen Li, Jianming Du, Shengxiang Zhang, Xuefeng Chen

**Affiliations:** ^1^College of Food Science and Engineering, Gansu Agricultural University, Lanzhou, China; ^2^School of Food and Biological Engineering, Shaanxi University of Science and Technology, Xi'an, China

**Keywords:** *Pleurotus eryngii* polysaccharides, obesity, structural characterization, metabolic pathways, metabolic differences

## Abstract

**Objective:**

In this study, the structure of *Pleurotus eryngii* polysaccharides (PEPs) was characterized, and the mechanism of PEP on obesity and hyperlipidemia induced by high-fat diet was evaluated by metabonomic analysis.

**Methods:**

The structure of PEPs were characterized by monosaccharide composition, Fourier transform infrared spectroscopy and thermogravimetry. In animal experiments, H&E staining was used to observe the morphological difference of epididymal adipose tissue of mice in each group. Ultrahigh performance liquid chromatography (UHPLC)-(QE) HFX -mass spectrometry (MS) was used to analyze the difference of metabolites in serum of mice in each group and the related metabolic pathways.

**Results:**

The PEPs contained nine monosaccharides: 1.05% fucose, 0.30% arabinose, 17.94% galactose, 53.49% glucose, 1.24% xylose, 23.32% mannose, 1.30% ribose, 0.21%galacturonic acid, and 1.17% glucuronic acid. The PEPs began to degrade at 251°C (T0), while the maximum thermal degradation rate temperature (Tm) appeared at 300°C. The results histopathological observation demonstrated that the PEPs had signifificant hypolipidemic activities. After PEPs intervention, the metabolic profile of mice changed significantly. A total of 29 different metabolites were selected as adjunctive therapy to PEPs, for treatment of obesity and hyperlipidemia-related complications caused by a high-fat diet. These metabolites include amino acids, unsaturated fatty acids, choline, glycerol phospholipids, and other endogenous compounds, which can prevent and treat obesity and hyperlipidemia caused by a high-fat diet by regulating amino acid metabolism, fatty acid metabolism, and changes in metabolic pathways such as that involved in the citric cycle (TCA cycle).

**Conclusions:**

The presented results indicate that PEPs treatment can alleviate the obesity and hyperlipidemia caused by a high-fat diet and, thus, may be used as a functional food adjuvant, providing a theoretical basis and technical guidance for the prevention and treatment of high-fat diet-induced obesity and hyperlipidemia.

## 1. Introduction

*Pleurotus eryngii* (*P. eryngii*) is a fungus with both food and medicinal uses (1–3). It is rich in nutrients, and is generally cheaper than other medicinal fungi. The raw materials are easy to obtain, and it is very popular among various populations. It has been reported to be cultivated in Europe, the Middle East, and North America, as well as in many parts of Asia (4–8); in particular, it has been widely cultivated in China (9). It has rich nutritional value and includes a variety of active substances, such as polysaccharides, polyphenols (10, 11), proteins, minerals, dietary fiber, vitamins, and so on (9, 12–15). *Pleurotus eryngii* polysaccharides (PEPs) have been reported to have many effects, such as antitumor, antibacterial, antiviral, diabetes-preventing, and immunity-improving effects (15–17). Polysaccharides can inhibit the weight of obese mice and regulate the levels of hypolipidemic metabolism such as triglycerides (TG), total cholesterol (TC), low-density lipoprotein cholesterol (LDL-C), high-density lipoprotein cholange (HDL-C), and free fatty acids (FFA), however, research on the associated functional mechanisms is not deep enough, especially relevant research on the anti-obesity and blood lipid-lowering mechanisms (18–21).

In recent years, obesity has led to a series of health problems worldwide; for example, obesity can cause high blood pressure, hyperlipidemia, hyperglycemia, atherosclerosis, coronary heart disease, and other cardiovascular and cerebrovascular diseases. At present, the most-commonly used anti-obesity drugs on the market are all chemically synthesized drugs, the long-term use of which can lead to toxic side-effects, Therefore, the development and research of natural active substances to replace synthetic drugs to prevent and assist in the treatment of diseases caused by obesity is considered necessary (22–24). In particular, research on the anti-obesity mechanisms of natural products is expected to be helpful in addressing the problems caused by obesity at their root.

In this work, we describe polysaccharides extracted from the fruit bodies of *P. eryngii*. First, infrared spectroscopy, thermal stability, and monosaccharide composition analyses of the *P. eryngii* polysaccharides (PEPs) were conducted. Then, through *in vivo* experiments in mice using LC-MS technology to analyze the differences in serum metabolites of different groups after the PEP intervention, we determined the changes in characteristic metabolic markers and related metabolic pathways, thus clarifying the anti-obesity mechanism of the PEPs from a metabolomics perspective. The presented results lay a theoretical and technical foundation for follow-up research on and the application of PEPs.

## 2. Materials and methods

### 2.1. Materials and reagents

The fresh fruiting bodies of *P. eryngii* were purchased from Xi'an of China. Fucose, arabinose, galactose, glucose, xylose, mannose, fructose, ribose, glucuronic acid, and dextran were purchased from Sigma Aldrich (St. Louis, Missouri) as monosaccharide standards. Chromatography-grade reagents were used in HPGPC and GC-MS analyses, as well as analytical-grade reagents throughout all experiments.

### 2.2. PEP preparation

We first cut the *P. eryngii* fruiting bodies into pieces, dried them until all water was removed, and crushed and filtered them using a 40 mesh sieve (13).

Polysaccharides were extracted by water extraction and alcohol precipitation (25). Through deproteinization by the Sevag method and freeze-drying, the PEPs were obtained. The total sugar and protein contents were about 67.8% (w/w) and 4.42% (w/w), respectively.

### 2.3. Structural characterization of PEP

#### 2.3.1. Monosaccharide composition analysis of PEP

Ion exchange chromatography (ICS) was conducted to analyze the monosaccharide composition of PEP. We accurately weighed 5 ± 0.05 mg of polysaccharide sample, put it into a clean chromatographic tube, added 2.5 M trifluoroacetic acid (TFA) solution, and heated the mixture at 121 °C for 2 h. The sample was dried under a nitrogen flow and washed with methanol 2–3 times. The dried PEPs sample was dissolved in 1 mL sterile water and diluted by 100 times, followed by amperometric detection. Then, 25 μL was taken and transferred to a chromatographic flask, which was detected under pulsed amperometric detection (PAD) conditions. The test mobile phase consisted of sodium hydroxide and sodium acetate, the flow rate was 1.0 mL min^−1^, the column temperature was 30°C, and the analytical column type was a CarboPac PA10. Previous studies have reported this method (26).

#### 2.3.2. FT-IR spectroscopy analysis

A total of 1 mg of the PEP sample and KBr powder were mixed, ground thoroughly, and pressed into a 1 mm thin plate. The plate was placed into a Bruker Vector 22 spectrometer (Bruker), and the FT-IR optical spectrum of the PEP was measured in the range of 4,000–400 cm^−1^ (27).

#### 2.3.3. Thermogravimetry of PEP

Next, 3 mg of PEP powder sample was weighed, and the TGA and DSC of the PEP was analyzed using an STA449 thermogravimetric analyzer (Bruker, Germany). The test conditions were under a nitrogen atmosphere with a temperature of 30–700°C, and the speed was gradually increased (10 K min^−1^) (28).

### 2.4. Animal experiments

Animal experiments were completed at Xi'an Medical College, and were approved by the Xi'an Medical College Laboratory Animal Care and Use Management Committee. The treatment of experimental animals was carried out in strict accordance with the Guide to the Care and Use of Laboratory Animals, formulated by the National Institutes of Health.

A total of 60 4-week-old (20 ± 2 g) male Kunming mice were purchased from Xi'an Jiaotong University (Xi'an, China). Each five mice were kept in a cage under the conditions of 22 ± 2 °C, 50–60% humidity, and 12-h light/dark cycle. The drinking water and standard rodent food of animals were provided *ad libitum* during the breeding period.

After 1 week of adaptive feeding, each group of 10 mice was randomly divided into 6 groups; namely, the normal control group (NC), hyperlipidemia model control group (MOD), positive group (PC), and three different PEP dose (50, 100, and 200 mg/kg/day) groups. In the first 8 weeks, the NC group was fed a normal diet, while other groups were fed a high-fat diet. In the last 8 weeks, except for the same diet as before, the NC and MOD groups were fed 0.1 mL of normal saline every day, the PC group was fed 0.1 mL of simvastatin every day, and the PEP groups were fed different doses of PEPs (50, 100, and 200 mg/kg/day) at 0.1 mL/10 g/day (29). The main formula of the high-fat feed was: 200 g casein, 3 g L-cysteine, 72.8 g corn starch, 25 g soybean oil, 177.5 g lard, 10 g mineral mixture S10026, 13 g dicalcium phosphate, 5.5 g calcium carbonate, 16.5 g potassium citrate, 10 g vitamin mixture V10001, and 2 g tartrate bile base.

### 2.5. Adipose histological morphology examinations

The histomorphology of mouse epididymal adipose tissue was observed using an Olympus micrography operating system of Japan.30 after H&E staining (30).

### 2.6. Untargeted serum metabolomic analysis

#### 2.6.1. Metabolite extraction

Ultrahigh-performance liquid chromatography (UHPLC)–(QE) HFX–mass spectrometry (MS) was carried out to evaluate the serum metabolic profiles of NC, MOD, PEP, and PC group mice. Serum samples were thawed at 4°C. Then, 50 μL of serum was accurately measured and transferred to a clean centrifuge tube. Then, 200 μL of methanol was added to the centrifuge tube, which was then vortexed. After mixing for 60 s to dissolve the sample as completely as possible, it was incubated on ice for 10 min at 4°C, then centrifuged for 10 min at 13,000 rpm. The supernatant was transferred to a glass insert for subsequent analysis (31).

#### 2.6.2. Liquid chromatography–MS analysis

The chromatographic determination parameters were set as: 100 × 2.1 mm, 1.9 μm. A Thermo Hypersil Gold C18 chromatographic column was used. The column temperature was 40°C, the sample chamber temperature was 4°C, the flow rate was 0.3 mL/min, and the sample injection volume was 4 μL. Mobile phase A was 0.1% formic acid aqueous solution, while acetonitrile and 0.1% formic acid solution were set as mobile phase B. Gradient elution was performed according to the following elution procedure: 0% B, 0–2 min; 0–15% B, 2–10 min; 15–30% B, 10–14 min; 30–95% B, 14–17 min; 95% B, 17–19 min; 95–100% B, 19–20 min, and 5 min for re-balancing.

The mass spectrometry conditions were as follows: electrospray ionization (ESI) was used in Negative ion mode, the scanning range was 75–1,125 m/z, the capillary voltage was 3.5 kV, the cone voltage was 35 kV, the ion source temperature was 280°C, the desolvation temperature was 450°C, the desolvation gas flow rate was 900 L/h, and the drying gas flow rate was 11 L/min.

### 2.7. Data processing

We used the LC-MS instrument's proprietary software (Compound Discover software) to extract and align the raw data obtained by LC-MS, and exported the sample information, retention time, mass-to-charge ratio, signal intensity, and so on, as a data set. The time bias in peak alignment was set to 60 s, and the Mass bias was set to 3 ppm.

ANOVA was used to evaluate the statistical differences, and Duncan's *Post-hoc* Test (SPSS17.0) was used to determine the significant differences between the test groups. We used the Compound Discover software of the Thermo Fisher LC-MS to process the original metabolic data, and imported the data into the SIMCA software for analysis.

## 3. Results

### 3.1. Characterization of PEPs

#### 3.1.1. Monosaccharide compositions of PEPs

**Figure 1:**
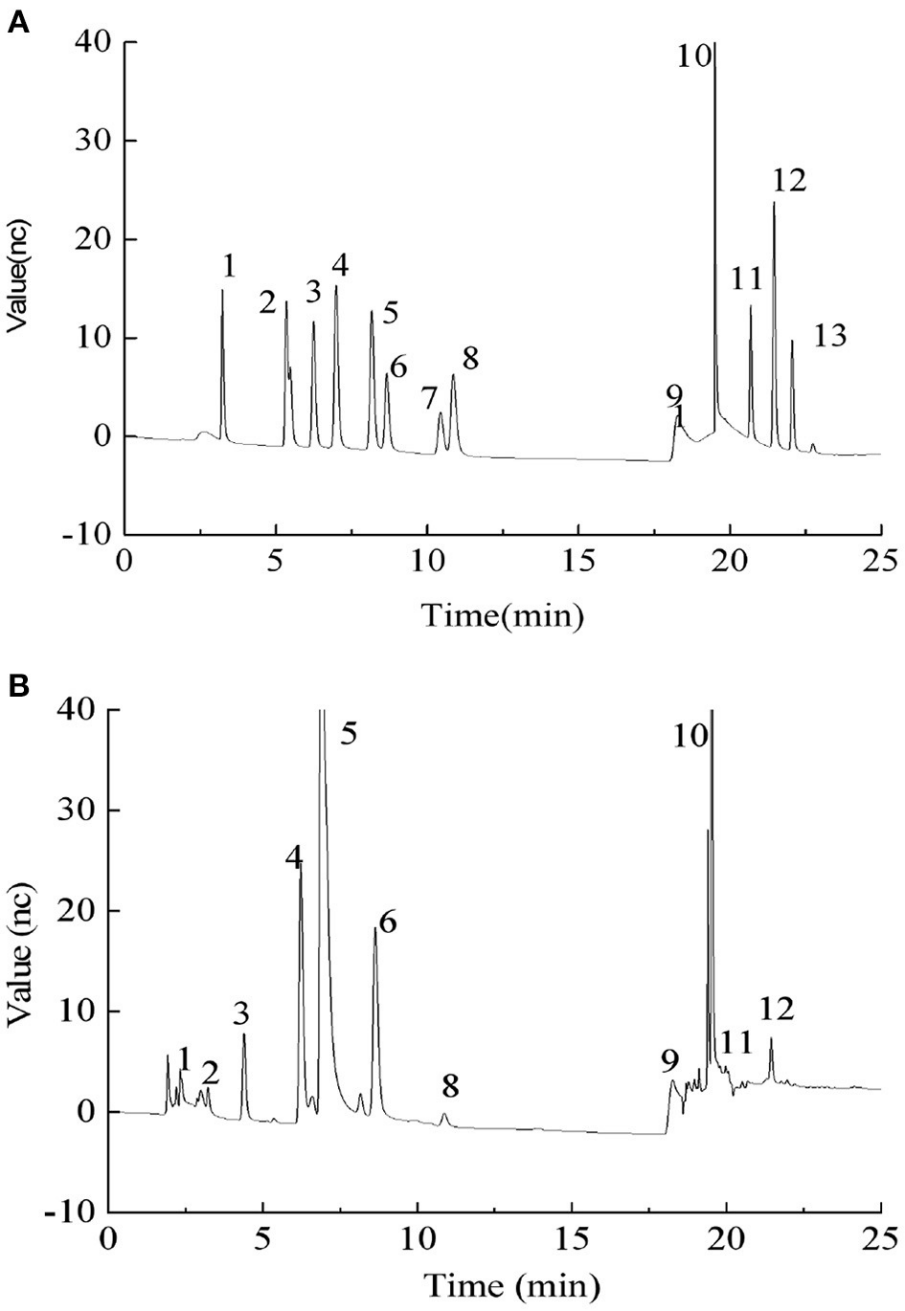
**(A)** Ion chromatography (ICS) profiles of monosaccharide standards. Monosaccharide standards 1–13 were as follows: (1) fucose, (2) arabinose, (3) galactose, (4) glucose, (5) xylose, (6) mannose, (7) fructose, (8) ribose, (11) galacturonic acid, (12) glucuronic acid, (13) mannuronic acid, and (9 and 10) the elution solvent; **(B)** Monosaccharide composition of PEPs.

We conducted ICS to determine the monosaccharide composition of PEPs, as shown in Figure 1. Compared with the standard, the PEPs mainly consisted of nine monosaccharides: fucose, arabinose, galactose, glucose, xylose, mannose, ribose, galacturonic acid and glucuronic acid, with percentages of 1.05, 0.30, 17.94, 53.49, 1.24, 23.32, 1.30, 0.21, and 1.17%, respectively.

#### 3.1.2. FT-IR spectroscopy analysis

**Figure 2:**
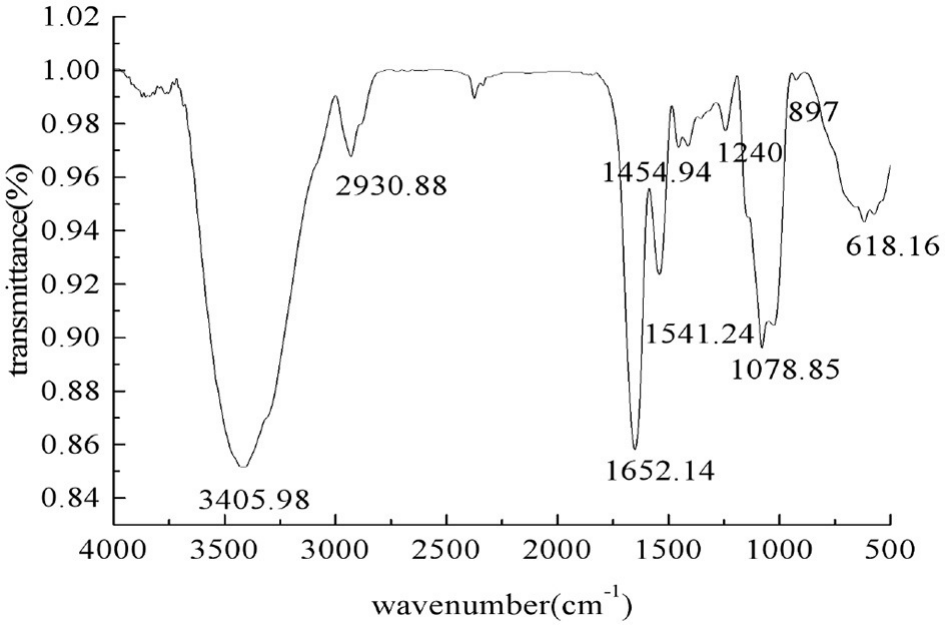
The IR spectrum of the PEPs.

The infrared spectrum of the PEPs is shown in Figure 2. A strong absorption peak of O–H can be observed at 3,405.98 cm^−1^, which is the characteristic absorption peak of sugar, indicating hydrogen bonding between molecules. There was a tensile vibration peak of C–H at 2,930.88 cm^−1^, and the peak at 1,652.14 cm^−1^ denotes the C=O tensile vibration peak. The absorption peaks at about 1,621 and 1,431 cm^−1^ indicated that the PEPs contained carboxyl groups. The wide absorption band with strong intensity at about 1,420–1,374 cm^−1^ in the PEP spectrum might be caused by the deformed vibration of C–H bonds. The absorption peaks at 1,240 and 897 cm^−1^ indicated that there was a sulfur ${\text{SO}}_{3}^{-1}$ group in the PEPs, including asymmetric S=O tensile vibration and symmetric C–O–S tensile vibration related to the C–O–SO_3_ group. The peak at 1,078.85 cm^−1^ indicated C–O–C tensile vibration. The absorption peak at about 570 cm^−1^ denoted tensile vibration of the pyran ring, indicating the presence of pyranose in the PEPs.

#### 3.1.3. Thermal stability of PEPs

**Figure 3:**
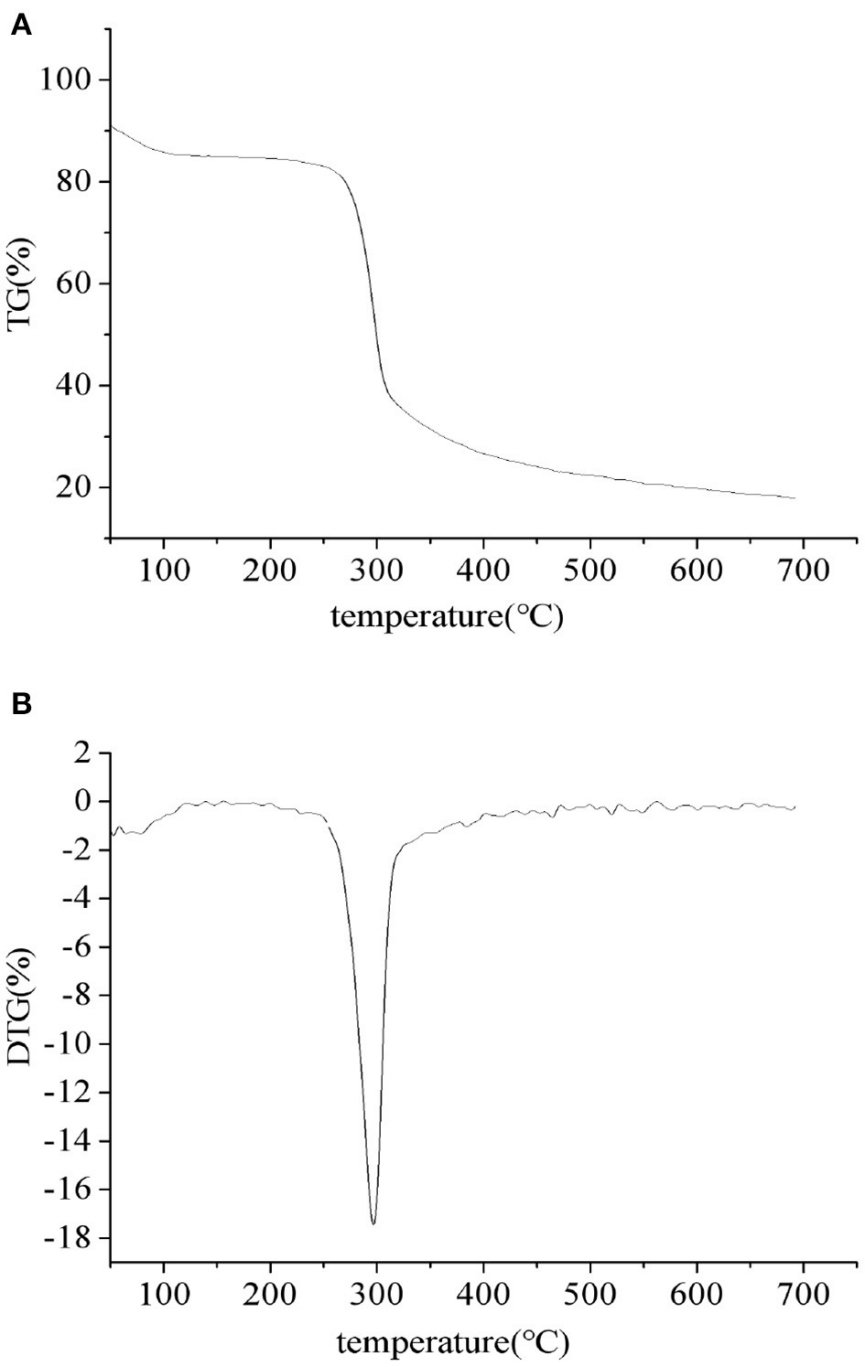
PEP TGA **(A)** and DTG **(B)** curves.

The thermal properties of the PEPs were investigated using TGA and DTG, as depicted in Figure 3. From the TGA (Figure 3A) and DTG (Figure 3B) curves, it can be seen that the thermal degradation process of the sample was divided into two stages, where the first stage was related to the evaporation of bound water in the sample and had nothing to do with the thermal degradation temperature of the polymer. Therefore, we did not further analyze the first stage of the TGA curve. In the second stage, the PEPs began to degrade at 251°C (T_0_), while the maximum thermal degradation rate temperature (T_m_) appeared at 300°C, caused by the breakage of glucosidic and hydrogen bonds due to demethoxylation and dehydration.

### 3.2. Variation of body weight and serum biochemical parameters

The changes in body weight and serum biochemical parameters of the different groups have been previously reported in our article “Optimization of extraction parameters of PEPs and evaluation of the hypolipidemic effect,” published in *RSC advances*.

### 3.3. Effects of PEP on adipose tissue histopathology

**Figure 4:**
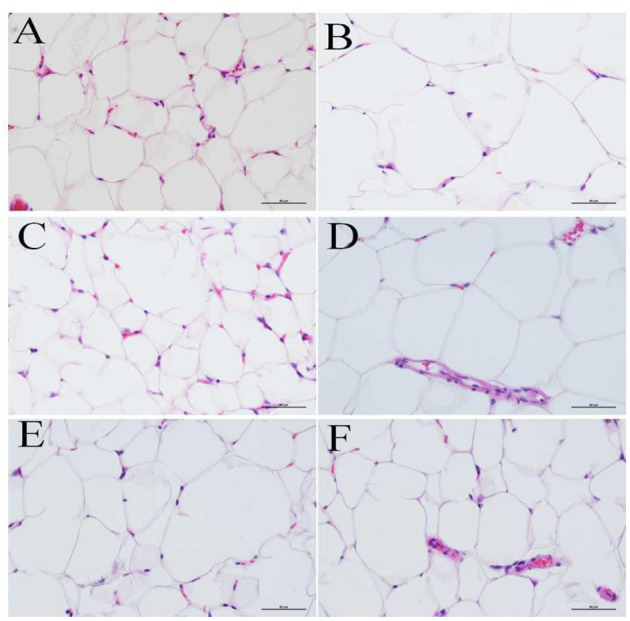
Morphological observations of fat tissue in different groups of mice (hematoxylin–eosin staining, 400×). **(A)** Normal control (NC) group; **(B)** Model control (MOD) group; **(C)** Positive control (PC) group; **(D)** Low-dose *Pleurotus eryngii* polysaccharide (LPEP) group; **(E)** Medium-dose *Pleurotus eryngii* polysaccharide (MPEP) group; and **(F)** High-dose *Pleurotus eryngii* polysaccharide (HPEP) group.

Epididymal adipose tissue was fixed with 4% paraformaldehyde, paraffin sectioned, and H&E stained. The morphology of testicular adipocytes was observed under a 400-fold optical microscope. The nucleus was stained blue by the basic dye (hematoxylin), while the cytoplasm was stained red with the acidic dye (eosin). During the staining process, the organic solvent can dissolve the lipid droplets inside the adipocytes, such that the adipocytes are vacuolated, while the nuclei are oblate, as they are squeezed to one side. Figures 4A–F shows the adipocyte morphology of mice in the NC, MOD, PC, HPEP, MPEP, and LPEP groups. It can be seen, from Figure 4, that there were differences in the volume and number of adipocytes in the different treatment groups. The volume of adipocytes in the MOD group was larger, while the number of small- and medium-sized adipocytes in a single field of view was lower. Comparing the PEP groups and the MOD group, we found that the PEP groups could reduce the volume of large adipocytes and increase the number of medium-sized adipocytes, where the effect in the HPEP group was the most obvious. The effect in the MPEP group was second to that in the HPEP group, while the LPEP group presented the lowest effect.

### 3.4. Non-targeted serum metabolomic analysis

#### 3.4.1. Multi-variate statistical analysis using PCA and PLS-DA

**Figure 5:**
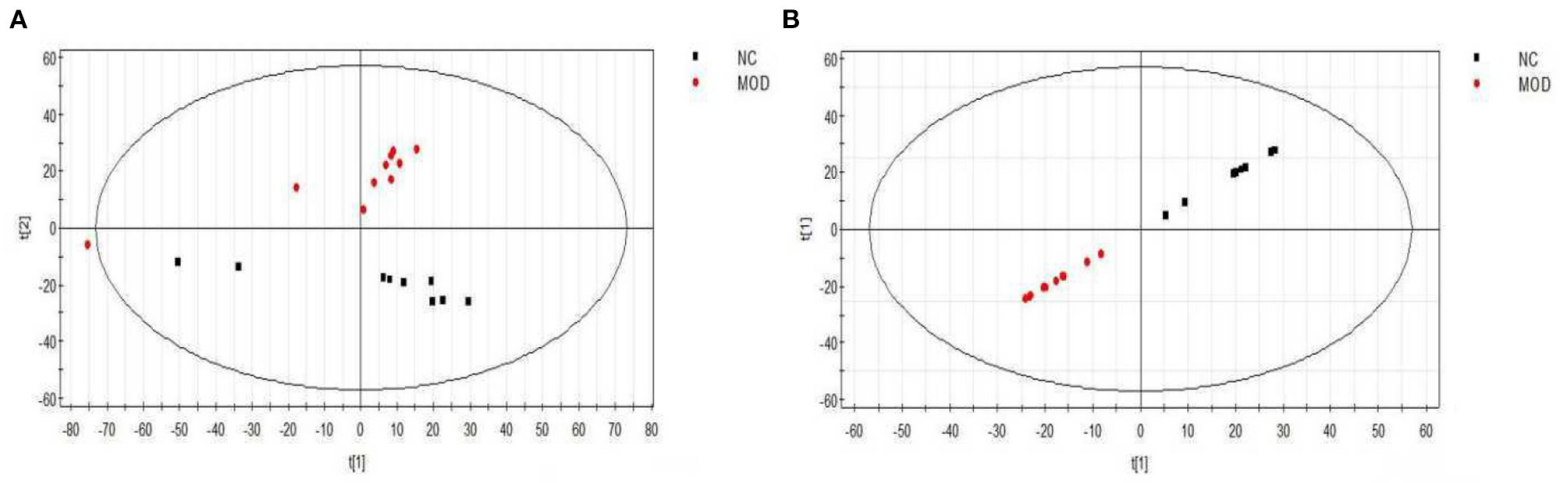
PCA **(A)** and PLS-DA **(B)** scores of serum samples from different groups of mice (positive). NC, Normal control group; MOD, model group.

As shown in Figure 5, both the PCA score map (2A) and the PLS-DA score map (2B) showed that the sample points for the normal diet group and the high-fat model group were separated, with sample points of the same color showing that the aggregation effect was good, within a certain range. The results indicated that there were obvious differences in serum metabolic components between the NC group and the MOD group, such that the high-fat model could be considered successful.

**Figure 6:**
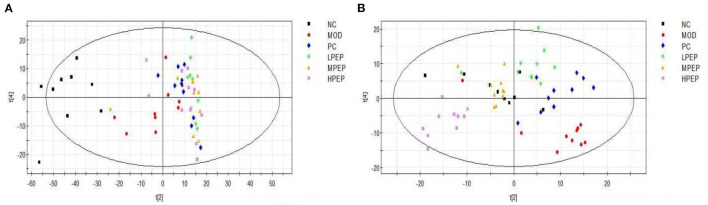
PCA **(A)** and PLS-DA **(B)** scores of serum samples from different groups of mice (positive); NC, Normal control group; MOD, model group; PC, positive control group; LPEP, low-dose polysaccharide group (50 mg/kg); MPEP, medium-dose polysaccharide group (100 mg/kg), (and HPEP, high-dose polysaccharide group (200 mg/kg).

Figure 6A shows the PCA scores for the NC, MOD, PC, LPEP, MPEP, and HPEP groups. It can be seen, from the figure, that the sample points for the NC group and other groups were far apart, indicating that the serum metabolites of the mice in the MOD group were significantly altered, compared with the NC group. The MOD group and the PEP intervention group were not completely separated, and there was some overlap, indicating that the PEPs has a certain effect on hypolipidemia. The PC group was also not separated from the PEP intervention group, indicating that the PEPs also had a lipid-lowering effect, with effect close to that of simvastatin. The sample points of the LPEP, MPEP, and HPEP groups were clustered together and not separated, indicating that the differences in serum metabolism between the different doses of *Pleurotus eryngii* polysaccharide groups were not obvious.

Figure 6B shows the principal component analysis PLS-DA scores for the NC, MOD, PC, LPEP, MPEP, and HPEP groups. It can be seen, from the figure, that the sample points between the MOD group and the NC, PC, LPEP, MPEP, and HPEP groups were relatively well-separated, indicating that the serum metabolites presented differences between the PEP groups and the MOD group, such that the PEP groups had a certain effect in lowering blood lipids and preventing obesity.

#### 3.4.2. Serum metabolite profiling and potential biomarker discovery

**Figure 7:**
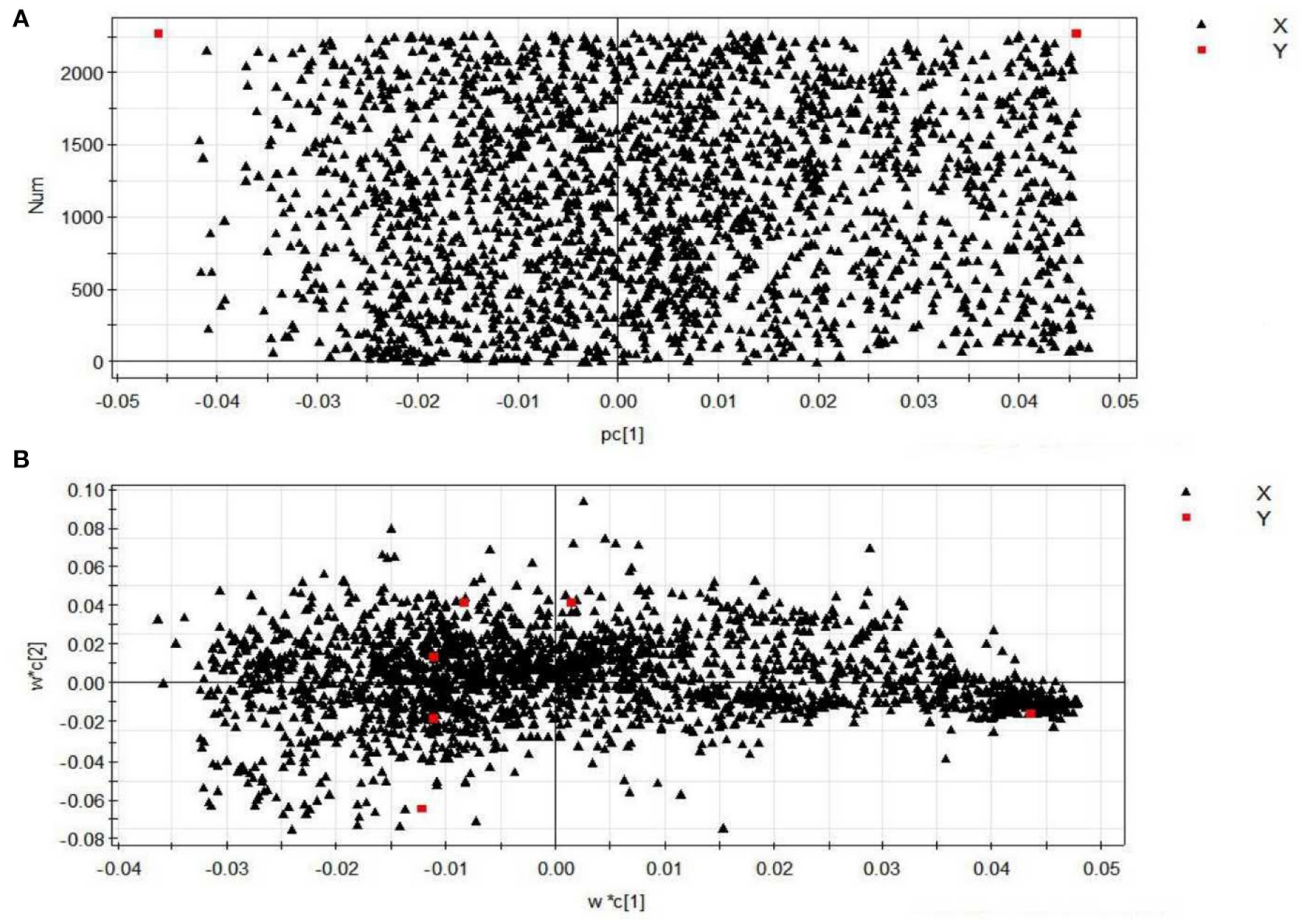
Loading plots of serum samples from different groups of mice (positive): **(A)** normal control group (NC)–model group (MOD) mouse serum sample loading diagram; **(B)** different doses of PEP group–positive control group–high fat model group mouse serum sample loading diagram.

The contributions of eigenvalues in the loading matrix represent the distance between the distribution points in the figure and the central axis. The farther the distance, the greater the contribution. The scattered points are most likely to indicate the metabolism of difference substances after PEP acted in obese mice. Figure 7A shows the loading matrix diagram for the NC and MOD groups. It can be seen, from the figure, that most of the points were far from the coordinate axes, indicating that there were differences in metabolized substances in the sera of NC and MOD group mice. Figure 7B shows the loading matrix diagram for the NC, MOD, PC, LPEP, MPEP, and HPEP groups. It can be seen, from the figure, that less points were scattered around the edges, thus being further from the coordinate axes, indicating that there were more differential metabolites in the sera of mice in the NC, MOD, PC, LPEP, MPEP, and HPEP groups. These substances might be potential metabolites for exploring and elucidating the pathogenesis of and drug therapy for hyperlipidemia and obesity-related complications.

Substances with VIP > 1.5 and *p* < 0.05 were considered as metabolic differential substances. The identified metabolic differences are listed in Table 1, with 29 substances most likely to be potential biomarkers having been screened. The table reflects the molecular weight of the metabolites, as well as the associated change trends and pathways. These metabolites include amino acids (e.g., arginine, citrulline, valine, and proline), unsaturated fatty acids (e.g., oleic acid, linoleic acid, and arachidonic acid), choline, lipid variants, and other endogenous compounds. The results demonstrated that the metabolic function of mice in the MOD group was disordered and the liver function was damaged, while the physiological function of the mice in the PEP intervention groups was restored, which might be related to the changes in the content of intercellular signaling molecules involved in processes such as lipid metabolism. These metabolic differences might allow for clarification of the pathogenesis of obesity-induced hyperlipidemia and complications, as well as the key points regarding the protective mechanism of the PEPs on obesity-induced hyperlipidemia and complications.

**Table 1:** Results of serum metabolite analysis in positive ion mode.

**Number**	**Mass**	**Name**	**Formula**	**Related pathway**	**MOD/** **NC**	**PC/** **MOD**	**LPEP/** **MOD**	**MPEP/** **MOD**	**HPEP/** **MOD**
1	517.32	LysoPC [18:3(9Z,12Z,15Z)]	C_26_H_48_NO_7_P	Fatty acid metabolism	S^#^	J^*^	J^*^	J^*^	J^*^
2	785.592	PC	C_44_H_84_NO_8_P	Glycerophospholipid metabolism	S^#^	J^*^	J^*^	J^*^	J^*^
3	117.072	Valine	C_5_H_11_NO_2_	Pantothenate and CoA biosynthesis	J^#^	J	S^*^	S^*^	S^*^
4	103.10	Choline	C_5_ H_13_NO	Glycine, serine, and threonine metabolism	S^#^	S	J^*^	J^*^	J^*^
5	129.08	Vigabatrin	C_6_ H_11_NO_2_	Fatty acid metabolism	S^#^	J	S^*^	S^*^	S^*^
6	131.08	Creatine	C_4_H_9_N_3_O_2_	Creatine metabolism	S^#^	S	S^*^	S^*^	S^*^
7	146.07	Glutamine	C_5_H_10_N_2_O_3_	Arginine biosynthesis	S^#^	J	J	J	J
8	174.11	Arginine	C_6_H_14_N_4_O_2_	Arginine and proline metabolism	S^#^	J^*^	J^*^	S^*^	S^*^
9	132.09	Ornithine	C_5_H_12_N_2_O_2_	Arginine biosynthesis	J^#^	S^*^	J	J	S^*^
10	115.06	Proline	C_5_H_9_NO_2_	Arginine and proline metabolism	J^#^	S^*^	J	J	S^*^
11	569.35	LysoPC (22:5)	C_30_H_52_NO_7_P	Fatty acid metabolism	S^#^	S^*^	S^*^	S^*^	S^*^
12	541.32	LysoPC (20:5)	C_28_ H_48_NO_7_P	Fatty acid metabolism	S	J	J	J	J
13	155.07	Histidine	C_6_H_9_N_3_O_2_	Histidine metabolism/beta-Alanine metabolism	S^#^	S^*^	S^*^	S^*^	S^*^
14	283.29	Stearamide	C_18_H_37_NO	Fatty acid metabolism	S^#^	J^*^	J	J^*^	J^*^
15	571.36	LysoPC (22:4)	C_30_H_54_ NO_7_P	Fatty acid metabolism	J^#^	S^*^	S^*^	S^*^	S^*^
16	175.10	L-Citrulline	C_6_H_13_N_3_O_3_	Arginine and proline metabolism	J^#^	S^*^	S^*^	S^*^	S^*^
17	304.24	Arachidonic acid	C_20_H_32_O_2_	Biosynthesis of unsaturated fatty acids	J^#^	S^*^	S^*^	S^*^	S^*^
18	125.01	Taurine	C_2_H_7_NO_3_S	Taurine and hypotaurine metabolism	J^#^	S^*^	S^*^	S^*^	S^*^
19	1586.10	Cytidine 5'-diphosphocholine	C_88_H_156_N_5_O_15_PS	Pyrimidine metabolism	S^#^	J^*^	J^*^	J^*^	J^*^
20	282.26	Oleic acid	C_18_H_34_O_2_	Fatty acid metabolism	J^#^	J	J	J	J
21	147.05	L-Glutamic acid	C_5_H_9_NO_4_	D-Glutamine and D-glutamate metabolism	S^#^	J^*^	J^*^	J^*^	J^*^
22	577.41	LysoPC [22:1(13Z)]	C_30_H_60_NO_7_P	Fatty acid metabolism	S^#^	J^*^	J^*^	J^*^	J^*^
23	122.05	Nicotinamide	C_6_H_6_N_2_O	Nicotinate and nicotinamide metabolism	J^#^	S^*^	S^*^	S^*^	S^**^
24	283.29	Stearamide	C_18_H_37_NO	Aminoacyl-tRNA biosynthesis	J^#^	J	J^*^	J^*^	J
25	255.26	Hexadecanamide	C_16_H_33_NO	Fatty acid metabolism	S^#^	J^*^	J^*^	J^*^	J^*^
26	131.06	Aminolevulinic acid	C_5_H_9_NO_3_	Aminoacyl-tRNA biosynthesis	S^#^	J^**^	J^**^	J^**^	J^**^
27	168.03	Uric acid	C_5_H_4_ N_4_ O_3_	Purine metabolism	J^#^	S^*^	S^*^	S^*^	S^*^
28	119.03	Succinic acid	C_4_H_6_O_4_	Citrate cycle (TCA cycle)	J^#^	S^*^	S^*^	S^*^	S^*^
29	281.23	Linoleic acid	C_18_H_32_O_2_	Linoleic acid metabolism	J^#^	S^*^	S^*^	S^*^	S^*^

S denotes increased; J denotes decreased;

# denotes that MOD/NC presents significant difference (p ≦ 0.05);

** denotes that PC/MOD, LPEP/MOD, MPEP/MOD, HPEP/MOD present extremely significant differences (p ≦ 0.01);

* denotes that PC/MOD, LPEP/MOD, MPEP/MOD, HPEP/MOD present significant differences (p ≦ 0.05).

Many previous studies have shown that lipid metabolism is related to many biological functions, being essential for many biochemical reactions (32). It has been found that the levels of unsaturated fatty acids (e.g., linoleic acid and arachidonic acid) were significantly down-regulated in a high-fat-induced model group, suggesting enhanced peroxidation and oxidative stress (33). Analysis of liver tissue sections has confirmed that peroxidation and oxidative stress could decompose apolipoprotein B protein, thereby weakening the secretion of very low-density lipoprotein (VLDL), reducing the output of liver TG and facilitating the accumulation of TG in the liver (34). Lipid β-oxidation generated a large number of electrons in the mitochondrial respiratory chain, producing excessive reactive oxygen species (ROS) (35). Linoleic acid is a polyunsaturated fatty acid that cannot be synthesized in the body and must be obtained from dietary sources (36). It has been reported that linoleic alcohol is related to atherosclerosis and inflammatory diseases, being a main component of the cell plasma membrane and a precursor to fatty acids such as prostaglandins and leukotrienes (37). In general, the decreased level of unsaturated fatty acids observed in this study indicated that a long-term high-fat diet might lead to hyperlipidemia, altered fatty acid metabolism, and oxidative stress reactions. It is necessary to further study the specific mechanisms involved in such lipid disorder processes.

Various amino acid metabolites are listed in Table 1, according to the observed changes in amino acid levels, including L-citrulline, taurine, and so on. L-citrulline is a naturally occurring non-essential amino acid, which is produced from arginine as a by-product of reactions catalyzed by the nitric oxide synthase (NOS) family. A study on hypercholesterolemia has shown that L-citrulline has favorable effects regarding the lowering of cholesterol and blood lipids, including lowering serum aspartate aminotransferase (AST) and alanine aminotransferase (ALT) levels, while HDL-C (38). It was found that the level of L-citrulline in hyperlipidemic mice was decreased, suggesting that the arginine metabolic pathway was disturbed. Our results indicated that PEP treatment could alleviate the abnormal arginine metabolism caused by HFD through the arginine metabolism pathway. Taurine is the most abundant amino acid in many tissues of humans and animals, and had many physiological functions, including bile acid coupling, antioxidant, and detoxification functions (39). Compared with the normal group, the level of taurine was decreased in the high-fat model group, which can be explained in two aspects: on one hand, taurine acts as an antioxidant to prevent oxidative stress from generating ROS; thus, acid binding promotes taurine secretion. On the other hand, the final product of cysteine catabolism is taurine. The high consumption of taurine in the model group indirectly reflected the significantly decreased cysteine content (40).

The tricarboxylic acid cycle (TCA) forms the hub of carbohydrate, lipid, and amino acid metabolic linkages (41), comprising the final metabolic pathway of these three nutrients; it is also the main pathway for glucose degradation and a key energy source for organisms. Succinic acid is an important intermediate metabolite in the TCA cycle, mainly presented in liver mitochondria (42). Lower levels of succinate were observed in the MOD group, while PEP treatment increased the level of succinate, compared to the MOD group; although not significantly.

Lysophosphatidylcholines (LysoPCs) are generated from the hydrolysis of oxidized phosphatidylcholines in LDL by phospholipase A2, and play various roles in many important biological processes (43). There is increasing evidence that hyperlipidemia refers to an imbalance in the lysozyme spectrum; for example, it has been reported that LysoPC (22:6) and LysoPC (20:4) were significantly reduced in mice fed a high-fat diet for 4 weeks whereas, in atherosclerotic rabbits, LysoPC (16:1), LysoPC (16:1), and LysoPC (20:5) levels were elevated. In this study, some lysophosphatidylcholines (LysoPCs), such as LysoPC [18:3(9Z,12Z,15Z)], LysoPC [20:5(5Z,8Z,11Z, 14Z, 17Z)], and LysoPC [22:1 (13Z)], were significantly up-regulated in the treatment group, compared with the normal diet group; LysoPC [22:5 (7Z, 10Z, 13Z, 16Z, 19Z)] was also up-regulated, but not significantly, compared with the NC group. In hyperlipidemic rats, these lysophosphatidylcholines (LysoPCs) exhibited different expression levels than in normal mice, suggesting that disturbances in glycerophospholipid metabolism might be involved in the pathogenesis of hyperlipidemia. LysoPC [18:3 (9Z, 12Z, 15Z)], LysoPC [20:5 (5Z, 8Z, 11Z, 14Z, 17Z)], and LysoPC [22:1 (13Z)] were significantly higher after PEP interference than in the model group, suggesting that the PEPs have a lipid-lowering effect and that the induced hyperlipidemia may be related to glycerophospholipid metabolism, which helped to improve our understanding of the mechanism underlying the effect of PEP treatment.

In cellular respiration, acetyl-L-carnitine is a transport and delivery tool that possesses the ability to transport fats while, at the same time, transporting fatty acids into mitochondria, thereby facilitating the production of ATP (44). Acetyl-L-carnitine can cross the blood–brain barrier, thus providing energy to brain cells. Carnitine, a quaternary ammonium compound, is present in the kidneys and liver, and mediates muscle transport in blood (45). The alteration of carnitine levels might have been related to the accumulation of serum TG levels in the model group.

The literature has suggested that the consumption of a high-fat diet could induce the excessive excretion of B vitamins (46). Nicotinamide (vitamin B3), as a component of coenzyme NAD, plays an important and complex function, and is catalyzed by purine riboside phosphatase to generate nicotinamide riboside. Nicotinamide riboside, a recently described natural NAD(+) precursor, has been reported to enhance oxidative metabolism and contribute to high-fat diet-induced metabolic abnormalities (47). Therefore, excessive excretion of nicotinamide can lead to a decrease in the yield of nicotinamide riboside, which was speculated to be the reason for the abnormal metabolism of mice in the MOD group induced by a high-fat diet in this study. We found that, compared with the normal diet group, the nicotinamide content in the high-fat model group was significantly decreased, while the nicotinamide content was significantly increased after PEP gavage.

#### 3.4.3. Metabolic pathways

**Figure 8:**
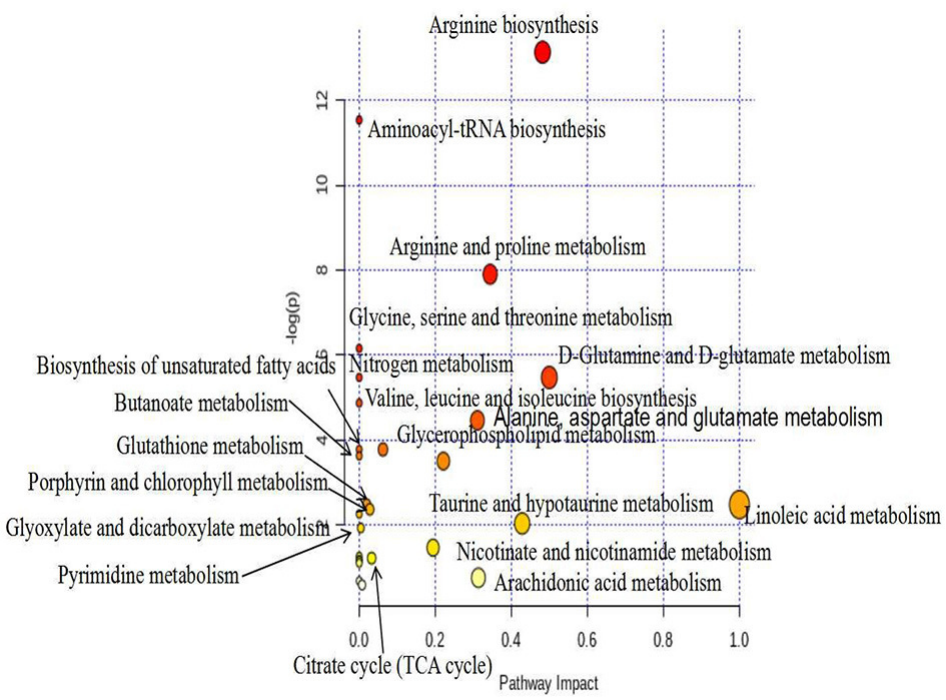
Metabolic pathway analysis (positive), metabolic pathway change diagram.

We uploaded the screened potential metabolic differences provided in Table 1 to the Metabo-Analyst (https://www.metaboanalyst.ca/Metabo-Analyst/home.xhtmL) database, using the Hypergeometric Test and out-degree centrality-based analysis method, in order to carry out enrichment analysis of related metabolic pathways and obtain the metabolic pathway change map, which is shown in Figure 8. Relevant pathways included glycerophospholipid metabolism; arachidonic acid metabolism; glycine, serine, and threonine metabolism; unsaturated fatty acid biosynthesis; pyrimidine metabolism; TCA cycle; histidine metabolism; and so on.

## 4. Discussion

Hyperlipidemia frequently occurs with high caloric intake in modern society, and is considered to be one of the highest risk factors for cardiovascular disease (48). Obesity may also lead to the occurrence of cardiovascular and cerebrovascular diseases, including hypertension, hyperlipidemia, hyperglycemia, atherosclerosis, coronary heart disease, and so on. Hyperlipidemic patients who take synthetic drugs for a long time in order to overcome such cardiovascular and cerebrovascular diseases may become dependent on these drugs, which can have certain toxic side-effects on the body. Therefore, the development of natural active substances to replace chemical synthetic drugs to treat the series of diseases caused by obesity is not only in line with the current dietary concept of medicine and food homology, but also serves to reduce the negative impacts of synthetic drugs on the human body (49). Polysaccharides extracted from the fruiting bodies of *P. eryngii* were utilized in *in vivo* experiments in mice, and the differences in serum metabolites between the different groups of mice after PEP intervention were analyzed by LC-MS. In this way, the relationships between characteristic metabolic markers and their related metabolic pathways were determined. Such changes could potentially elucidate the associated mechanism of action, providing significant theoretical value for the research and utilization of natural active substances.

In this study, the monosaccharide composition of the polysaccharides extracted from the fruiting bodies of *P. eryngii* was first analyzed. The monosaccharide composition of PEPs includes reducing sugars, they play a role in the antioxidant process of PEPs by increasing the production of antioxidant enzymes physical activity, reduce the content of MDA and reduce free radicals in the body, improve excessive oxidation of lipids, thereby regulating lipid metabolism (50). Fourier transform-infrared spectroscopy analysis demonstrated that the monosaccharide molecules were closely cross-linked through hydrogen bonds, such that the chemical bond structure was complex, and indicated the presence of pyranose in the PEPs. The thermal analysis of PEPs provides a temperature reference range for the degradation of polysaccharides into small sugars and the fracture of chemical bonds. In the animal experiment, 60 mice were divided into six groups (*n* = 10 per group), which were denoted as the NC, MOD, PC, LPEP, MPEP, and HPEP groups. The effect of PEP treatment on the histopathology of adipose tissue was observed by staining, and it was found that the MOD group had larger adipocyte volume, with the number of medium and small adipocytes in a single field being higher. Comparing the PEP and MOD groups, we found that the PEP group presented reduced volume of large adipocytes and increased number of medium adipocytes, where the HPEP group showed the most significant effect. The effect in the MPEP group was second only to that in the HPEP group, while the LPEP group had the lowest effect. Bederman et al. have found that small fat stores in cystic fibrosis mice were characterized by reduced cell volume, not cell number; the same conclusion was also found in this work (51).

Studies have shown that a high-fat diet can cause obesity in rats, as well as increasing serum total cholesterol and high-density lipoprotein cholesterol while reducing low-density lipoprotein cholesterol (i.e., abnormal blood lipid metabolism), resulting in hyperlipidemia. The liver is the key site of lipid metabolism. The relative content of blood lipids in the human body is mainly regulated by hepatocytes, which convert cholesterol into bile acids and secrete bile to promote the digestion and absorption of fat in the intestine (52, 53). When the liver function is damaged, the production and excretion of bile is hindered, resulting in abnormal lipid metabolism in the body. However, dietary fiber can reduce the intestinal absorption of cholesterol and, thus, reduce the content of serum total cholesterol and low-density lipoprotein cholesterol. Fungi are rich in proteins, crude fiber, and a large number of biologically active substances, such as polysaccharides and polyphenols. As described in this experiment, PEPs significantly inhibited the weight gain caused by a high-fat diet, thereby preventing obesity. They also reduced the accumulation of liver fat in rats fed a high-fat diet and, so, may prevent dyslipidemia by improving liver function, thus promoting liver metabolism. Dietary fiber can stimulate gastrointestinal motility, and may also be fermented in the intestine to generate metabolites such as short-chain fatty acids, thereby promoting the metabolism (54).

Hyperlipidemia model mice presented abnormal metabolism of blood lipids, manifested as high TC, TG, and LDL-C or low HDL-C. After intragastric administration of PEPs, the serum levels of TC and LDL-C in mice were significantly lower, the level of TG decreased, and that of HDL-C was increased. Cholesterol often accumulates in vascular endothelial cells, potentially causing arteriosclerosis in the body, and low-density lipoprotein-C in plasma is the main carrier of plasma cholesterol in the body. High-density lipoprotein cholesterol has important biological activity, helping to transport excess cholesterol from all parts of the body to the liver. This cholesterol was excreted from the body in feces through a series of reactions, indicating that PEP treatment could ultimately reduce blood lipids by promoting the decomposition of cholesterol (55, 56).

Using multivariate statistical analysis (i.e., PCA) in the non-targeted serum metabolomics analysis, we found that the serum metabolites of the mice in the MOD group were significantly altered, compared with the NC group. Meanwhile, the MOD group and the PEP intervention groups were not completely separated, with small overlap. This indicates that PEP treatment had a certain effect in terms of lowering blood lipids. Furthermore, the PC group was not separated from the PEP intervention group, indicating that PEP treatment provides a lipid-lowering effect, where the effect was significant. The sampling points of the LPEP, MPEP, and HPEP groups were basically clustered together and were not separate, indicated that different doses did not lead to significant differences in serum metabolites between the polysaccharide groups. Principal component analysis of PLS-DA scores demonstrated that the sampling points of the MOD group and those of the NC, PC, LPEP, MPEP, and HPEP groups were significantly separated, indicating differences in serum metabolites between the PEP and MOD groups, where the PEP groups had a certain effect on reducing blood lipids and preventing obesity-related effects. Serum metabolite analysis and potential biomarker analysis showed that there were metabolic differences in sera when comparing the NC and MOD groups. Meanwhile, when comparing the NC, MOD, PC, LPEP, MPEP, and HPEP groups, there were more differential metabolites in the sera of mice; these metabolite substances may have potential for exploration and elucidation of the pathogenesis of and drug treatment targeting hyperlipidemia and obesity-related complications. Finally, the metabolic pathway analysis indicated that, within the whole process, the pathways related to lymphoid phospholipid metabolism; arachidonic acid metabolism; glycine, serine, and threonine metabolism; unsaturated fatty acid biosynthesis; pyrimidine metabolism; TCA cycle; and histidine metabolism, among others, have certain importance, consistent with the findings of Hoxha et al. (57).

## 5. Conclusion

In this study, PEPs were characterized as containing nine main monosaccharides. Furthermore, they began to degrade at 251 °C (T_0_), while the maximum thermal degradation rate temperature (T_m_) appeared at 300 °C. Histopathological observation of epididymal adipose tissues indicated that the PEPs present hypolipidemic activities; as such, the hypolipidemic effect of PEP treatment in high-fat diet-induced mice was investigated. Serum metabolomic analyses demonstrated that the effects of PEP treatment involved the glycerophospholipid metabolism, fatty acid biosynthesis, pyrimidine metabolism, TCA cycle, and amino acid metabolism pathways, among others, and significantly improved obesity and hyperlipidemia-related complications. The results of this study provide insights into the possible pathways for PEPs as an adjunctive treatment for hyperlipidemia and obesity, as well as providing theoretical support for the design and production of hypolipidemic functional foods.

## Data availability statement

The raw data supporting the conclusions of this article will be made available by the authors, without undue reservation.

## Ethics statement

All animal treatments were conducted strictly in accordance with the National Institutes of Health Guidelines for the Care and Use of Laboratory Animals. Animal experiments were approved by the Administrative Committee of the Experimental Animal Care and Use at Xi'an Medical University.

## Author contributions

YZ: investigation, formal analysis, and writing—original draft. XC: writing—review, editing, and supervision. ZZ, LW, WL, JD, and SZ: investigation. All authors read and approved the final manuscript.

## References

[1] Singh U Gautam A Singha TK Tiwari A Tiwari P Sahai V. Mass production of *Pleurotus eryngii* mycelia under submerged culture conditions with improved minerals and vitamin d_2_. LWT. (2020) 131:109665. 10.1016/j.lwt.2020.109665

[2] Krakowska A Zieba P Włodarczyk A Kała K Sułkowska-Ziaja K Bernaś E. Selected edible medicinal mushrooms from Pleurotus genus as an answer for human civilization diseases. J Food Chemistry. (2020) 327:127084. 10.1016/j.foodchem.2020.127084 32446029

[3] Ma N Du H Ma G Yang W Han Y Hu Q. Characterization of the Immunomodulatory Mechanism of a *Pleurotus eryngii* Protein by Isobaric Tags for Relative and Absolute Quantitation Proteomics. J Agr Food Chem. (2020) 68:13189–99. 10.1021/acs.jafc.0c00219 32227945

[4] Barone R Bavisotto CC Rappa F Gargano ML Macaluso F Paladino L. Jnk pathway and heat shock response mediate the survival of c26 colon carcinoma bearing mice fed with the mushroom *Pleurotus eryngii* var. eryngii without affecting tumor growth or cachexia. Food Function. (2021) 12:3083–95. 10.1039/D0FO03171B 33720221

[5] Dos Santos TL Tavares OCH Lopes Sd Elias SS Berbara RLL García AC. Environmental implications of the organic matter structure for white-rot fungus *Pleurotus eryngii* growth in a tropical climate. Fungal Biol. (2021) 125:845–59. 10.1016/j.funbio.2021.05.006 34649671

[6] Teniou S Bensegueni A Hybertson BM Gao BF Bose K McCord JM. Biodriven investigation of the wild edible mushroom *Pleurotus eryngii* revealing unique properties as functional food. J Funct Foods. (2022) 89:104965. 10.1016/j.jff.2022.104965

[7] Zhang C Song XL Cui WJ Yang QH. Antioxidant and anti-ageing effects of enzymatic polysaccharide from *Pleurotus eryngii* residue. Int J Biol Macromol. (2021) 73:341–50. 10.1016/j.ijbiomac.2021.01.030 33434551

[8] Abreu H Zavadinack M Smiderle FR Cipriani TR Cordeiro LMC Iacomini M. Polysaccharides from *Pleurotus eryngii* : selective extraction methodologies and their modulatory effects on THP-1 macrophages. Carbohyd Polym. (2020) 252:0144–8617. 10.1016/j.carbpol.2020.117177 33183624

[9] Ma G Xu Q Du H Kimatu BM Su A Yang W. Characterization of polysaccharide from *Pleurotus eryngii* during simulated gastrointestinal digestion and fermentation. Food Chem. (2022) 370:131303. 10.1016/j.foodchem.2021.131303 34662794

[10] Hellen A Ribeiro SF Lanzi SG Sovrani V Cordeiro L Icomini M. Naturally methylated mannogalactans from the edible mushrooms *Pholiota nameko* and *Pleurotus eryngii*. J Food Compost Anal. (2021) 102:0889–1575. 10.1016/j.jfca.2021.103985

[11] Færestrand EC Winther WC Alistair W Frode R Berit SA. Water-soluble polysaccharides from *Pleurotus eryngii* fruiting bodies, their activity and affinity for Toll-like receptor 2 and dectin-1. J Carbohydrate Polymers. (2021) 264:117991. 10.1016/j.carbpol.2021.117991 33910729

[12] Kleftaki S-T Simati S Amerikanou C Gioxari A Tzavara C Zervakis G. *Pleurotus eryngii* improves postprandial glycaemia, hunger and fullness perception, and enhances ghrelin suppression in people with metabolically unhealthy obesity. Pharmacol Res. (2021) 175:105979. 10.1016/j.phrs.2021.105979 34798266

[13] Park Y-S Jang S Lee H Kang S Seo H Yeon S. Identification of the antidepressant function of the edible mushroom *Pleurotus eryngii*. J. Fungi. (2021) 7:190. 10.3390/jof7030190 33800437 PMC8000720

[14] Hu Q Yuan B Wu X Du H Gu M Han Y. Dietary intake of *Pleurotus eryngii* ameliorated dextran-sodium-sulfate-induced colitis in mice. Food Res. (2019) 63:1801265. 10.1002/mnfr.201801265 31125180

[15] Yang RL Li Q Hu QP. physicochemical properties, microstructures, nutritional components, and free amino acids of *Pleurotus eryngii* as affected by different drying methods Sci. Rep-UK. (2020) 10:121. 10.1038/s41598-019-56901-1 31924832 PMC6954260

[16] Wang T Yue S Jin Y Wei H Lu L. Advances allowing feasible pyrg gene editing by a crispr-cas9 system for the edible mushroom *Pleurotus eryngii*. Fungal Genet Biol. (2021) 147:103509. 10.1016/j.fgb.2020.103509 33400990

[17] Jf A Zheng WB Pei WC Ming WA. Extraction, structure and bioactivities of the polysaccharides from ginkgo biloba: a review. Int J Biol Macromol. (2020) 162:1897–905. 10.1016/j.ijbiomac.2020.08.141 32827622

[18] Ma G Kimatu BM Yang W Pei F Zhao L Du H. Preparation of newly identified polysaccharide from *Pleurotus eryngii* and its anti-inflammation activities potential. J. Food Sci. (2020) 85:2822–31. 10.1111/1750-3841.15375 32794226

[19] Acay H Yildirim A Güzel EE Kaya N Baran MF. Evaluation and characterization of *Pleurotus eryngii* extract-loaded chitosan nanoparticles as antimicrobial agents against some human pathogens. Prep Biochem Biotech. (2020) 50:1–10. 10.1080/10826068.2020.1765376 32420792

[20] Vetvickaa V Goverb O Karpovskyb M Hayby H Danay O Ezov N. Immune-modulating activities of glucans extracted from *Pleurotus ostreatus* and *Pleurotus eryngii*. J Funct Foods. (2019) 54:81–91. 10.1016/j.jff.2018.12.034

[21] Sua DB Lva WQ Wang Y Wang LJ Li D. Influence of microwave hot-air flow rolling dry-blanching on microstructure, water migration and quality of *Pleurotus eryngii* during hot-air drying. Food Control. (2020) 114:107228–107228. 10.1016/j.foodcont.2020.107228

[22] Jo K-J Ghim J Kim J Lee H Lee TG Kim J-I. Water Extract of *Pleurotus eryngii* var. ferulae prevents high-fat diet-induced obesity by inhibiting pancreatic lipase. J Med Food. (2019) 22:178–85. 10.1089/jmf.2018.4255 30657431

[23] Zhao YN Chen X Zhao Y Jia W Chang X Liu H. Optimization of extraction parameters of Pleurotus eryngii polysaccharides and evaluation of the hypolipidemic effect. RSC Advances. (2020) 10:11918–28. 10.1039/C9RA10991A 35694324 PMC9122559

[24] Nakahara D Nan C Mori K Hanayama M Kikuchi H Hirai S. Effect of mushroom polysaccharides from *Pleurotus eryngii* on obesity and gut microbiota in mice fed a high-fat diet. Eur J Nutr. (2020) 59:3231–44. 10.1007/s00394-019-02162-7 31865422

[25] Morales D Piris AJ Ruiz-Rodriguez A Prodanov M Soler-Rivas C. Extraction of bioactive compounds against cardiovascular diseases from Lentinula edodes using a sequential extraction method. Biotechnol Prog. (2018) 34:746–55. 10.1002/btpr.2616 29388355

[26] Liu G Chen H Chen J Wang X Gu Q Yin Y. Effects of bifidobacteria-produced exopolysaccharides on human gut microbiota *in vitro*. Appl Microbiol Biotechnol. (2019) 103:1693–702. 10.1007/s00253-018-9572-6 30569218

[27] Seedevi P Moovendhan M Sudharsan S Sivasankar P Sivakumar L Vairamani S. Isolation and chemical characteristics of rhamnose enriched polysaccharide from Grateloupia lithophila. Carbohydr Polym. (2018) 195:486–94. 10.1016/j.carbpol.2018.05.002 29805003

[28] Hsieh WH Cheng WT Chen LC. Lin, SY. Non-isothermal dehydration kinetic study of aspartame hemihydrate using DSC, TGA and DSC-FTIR microspectroscopy. Asian J Pharm Sci. (2018) 13:212–9. 10.1016/j.ajps.2017.12.001 32104394 PMC7032143

[29] Kilari BP Mudgil P Azimullah S Bansal N Ojha S Maqsood S. Effect of camel milk protein hydrolysates against hyperglycemia, hyperlipidemia, and associated oxidative stress in streptozotocin (STZ)-induced diabetic rats. J Dairy Sci. (2021) 104:1304–17. 10.3168/jds.2020-19412 33272578

[30] Chen X He Y Xu A Deng Z Feng J Lu F. Increase of glandular epithelial cell clusters by an external volume expansion device promotes adipose tissue regeneration by recruiting macrophages. Biosci Rep. (2019) 39:BSR20181776. 10.1042/BSR20181776 30760630 PMC6390125

[31] Pichini S Malaca S Gottardi M. UHPLC-MS/MS analysis of cannabidiol metabolites in serum and urine samples. Application to an individual treated with medical cannabis. Talanta. (2021) 223:121772. 10.1016/j.talanta.2020.121772 33298281

[32] Guo J Wu J Wei D Wang T Hu Y Lin Y. Association between greenness and dyslipidemia in patients with coronary heart disease: a proteomic approach. Ecotox Environ Safe. (2022) 231:113199. 10.1016/j.ecoenv.2022.113199 35042090

[33] Ma X Liao Z Li R Xia W Guo H Luo J. Myocardial injury caused by chronic alcohol exposure—a pilot study based on proteomics. Molecules. (2022) 27:4284. 10.3390/molecules27134284 35807529 PMC9268295

[34] Guo W-L Deng J-C Pan Y-Y Xu J-X Hong J-L Shi F-F. Hypoglycemic and hypolipidemic activities of Grifola frondosa polysaccharides and their relationships with the modulation of intestinal microflora in diabetic mice induced by high-fat diet and streptozotocin. Int J Biol Macromol. (2020) 153:1231–40. 10.1016/j.ijbiomac.2019.10.253 31759027

[35] Zhang W-L Williams D Onyia O Morselli M Pellegrini M Arnold M. Sex difference in congenital hereditary endothelial dystrophy and a Slc4a11-/- mouse model. Invest Ophth Vis Sci. (2022) 63:2283.

[36] Nasir Y Farzollahpour F Mirzababaei A Maghbooli Z Mirzaei K. Associations of dietary fats intake and adipokines levels in obese women - ScienceDirect. Clin Nutr ESPEN. (2021) 43:390–6. 10.1016/j.clnesp.2021.03.018 34024546

[37] Wu T Wang G Xiong Z Xia Y Song X Zhang H. Probiotics interact with lipids metabolism and affect gut health. Front Nutr. (2022) 9:917043. 10.3389/fnut.2022.917043 35711544 PMC9195177

[38] Danboyi T Alhassan AW Jimoh A Hassan-Danboyi E. Effect of L-citrulline supplementation on blood glucose level and lipid profile in high-fat diet - and dexamethasone-induced type-2 diabetes in male wistar rats. Niger J Exp Clin Biosci. (2022) 8:100–7. 10.4103/njecp.njecp_23_20

[39] Mehdi OM Reza H Vahid G Narges A Hossein N. Taurine treatment provides neuroprotection in a mouse model of manganism. Biol Trace Elem Res. (2019) 190:384–95. 10.1007/s12011-018-1552-2 30357569

[40] Hyun KS Hyeji S Doyoung K Yeon YD YoungSuk J. Taurine ameliorates tunicamycin-induced liver injury by disrupting the vicious cycle between oxidative stress and endoplasmic reticulum stress. Life. (2022) 12:354. 10.3390/life12030354 35330105 PMC8951380

[41] Ciccarone F Vegliante R Leo L Ciriolo MR. The TCA cycle as a bridge between oncometabolism and DNA transactions in cancer. Semin Cancer Biol. (2017) 47:50–6. 10.1016/j.semcancer.2017.06.008 28645607

[42] Hijaz F Killiny N. Exogenous GABA is quickly metabolized to succinic acid and fed into the plant TCA cycle. Plant Signal Behav. (2019) 14:e1573096. 10.1080/15592324.2019.1573096 30676228 PMC6422366

[43] Maslanka K Smolenska GS Michur H. Lysophosphatidylcholines: bioactive lipids generated during storage of blood components. Arch Immunol Ther Exp (Warsz). (2012) 60:55–60. 10.1007/s00005-011-0154-x 22167322

[44] Hsue TY Wang X Huang Y. 376 Effect of fatty acids on myogenesis and mitochondrial biosynthesis during murine skeletal muscle cell differentiation. J Anim Sci. (2017) 95:186. 10.2527/asasann.2017.376

[45] Jubie S Jawahar N Arigo A Prabha T Anjali PB. Stability enhancement and formulation development of l. J Drug Deliv Sci Technol. (2020) 55:103762. 10.1016/j.jddst.2019.101474

[46] Zheng Y Ma AG Zheng MC Wang Q-Z Liang H Han X. B vitamins can reduce body weight gain by increasing metabolism-related enzyme activities in rats fed on a high-fat diet. Curr Med Sci. (2018) 38:174–83. 10.1007/s11596-018-1862-9 30074168

[47] Carles C Houtkooper RH Pirinen E Youn DY Oosterveer MH Cen Y. The NAD+ precursor nicotinamide riboside enhances oxidative metabolism and protects against high-fat diet-induced obesity. Cell Metab. (2012) 15:838–47. 10.1016/j.cmet.2012.04.022 22682224 PMC3616313

[48] Nie Y Luo FJ Wang L Yang T Shi L Li X. Anti-hyperlipidemic effect of rice bran polysaccharide and its potential mechanism in high-fat diet mice. Food Funct. (2017) 8:4028–41. 10.1039/C7FO00654C 28869259

[49] Zhao Z Zhang Y Liu L Chen Y Wang D Jin X. Metabolomics study of the effect of smoking and high-fat diet on metabolic responses and related mechanism following myocardial infarction in mice. Life Sci. (2020) 263:118570. 10.1016/j.lfs.2020.118570 33058917

[50] Xu Y Zhang X Yan XH Zhang J Wang L Xue H. Characterization, hypolipidemic and antioxidant activities of degraded polysaccharides from *Ganoderma lucidum*. Int J Biol Macromol. (2019) 135:706–16. 10.1016/j.ijbiomac.2019.05.166 31129213

[51] Bederman I DiScenna A Henderson L Perez A Klavanian J Kovtun D. Small adipose stores in cystic fibrosis mice are characterized by reduced cell volume, not cell number. Am J Physiol-Gastr L. (2018) 315:943–53. 10.1152/ajpgi.00096.2017 30188751 PMC6336944

[52] Wang ZJ Su Y Li K LiuX. Hypolipidemic Effects of Polysaccharides from Fermented Seaweed. Mater Sci Eng C. (2019) 612:022070. 10.1088/1757-899X/612/2/022070

[53] Yoshida H Tsuhako R Sugita C Kurokawa M. Glucosyl hesperidin has an anti-diabetic effect in high-fat diet-induced obese mice. Biol Pharm Bull. (2021) 44:422–30. 10.1248/bpb.b20-00849 33642550

[54] Kim JI Yun JA Jeong YK Baek HJ. Hypoglycemic and hypolipidemic effects of samnamul (shoot of Aruncus dioicus var. kamtschaticus Hara) in mice fed a high-fat/ high-sucrose diet. Food Sci Biotechnol. (2018) 27:1467–73. 10.1007/s10068-018-0390-5 30319857 PMC6170272

[55] He X Zheng N He J Liu C Feng J Jia W. Gut microbiota modulation attenuated the hypolipidemic effect of simvastatin in high-fat/cholesterol-diet fed mice. J Proteome Res. (2017) 16:1900–10. 10.1021/acs.jproteome.6b00984 28378586 PMC5687503

[56] Eo H Park JE Jeon YJ Lim Y. Ameliorative effect of ecklonia cava polyphenol extract on renal inflammation associated with aberrant energy metabolism and oxidative stress in high fat diet-induced obese mice. J Agric Food Chem. (2017) 65:3811–8. 10.1021/acs.jafc.7b00357 28459555

[57] Hoxha B Zappacosta B Domi E Hoxha M. The interaction between arachidonic acid metabolism and homocysteine. Endocr Metab Immune Disord Drug Targets. (2021) 21:1232–41. 10.2174/1871530320999200904130504 32888285

